# Isolated Acquired Macrodactyly of the Left Fourth Toe

**DOI:** 10.7759/cureus.12648

**Published:** 2021-01-12

**Authors:** Leon Alexander, Ahmed Mohamed H. El Kazzaz, Michael Schenker

**Affiliations:** 1 Plastic & Reconstructive Surgery, Sheikh Khalifa Medical City, Abu Dhabi, ARE

**Keywords:** macrodactyly, overgrowth, amputation, soft tissue reduction, epiphysiodesis

## Abstract

Macrodactyly is a rare congenital disorder affecting the digits of hands and feet. It is often distressing for both parents and children. Surgical options range from soft tissue debulking with or without osteotomy and physeal arrest to simple or ray amputation. Although amputation is generally reserved for the severe progressive variant of macrodactyly and revision surgery, there is controversy regarding whether initial amputation or debulking is the best approach. We present a case of isolated macrodactyly of a minor toe, where debulking showed superior results.

## Introduction

Macrodactyly is a non-hereditary congenital disorder of the limbs encountered rarely in clinical practice. It accounts for less than 1% of all extremity congenital anomalies [1]. Tissue within the affected portions of the extremity show specific patterns of growth and anatomical relationships in contrast to neoplastic lesions. Nerve involvement has been consistently reported in macrodactyly and a biological basis of nerve-mediated overgrowth has been suggested. Sometimes there is an overgrowth of limbs due to associated fat accumulation and hypertrophy of skeletal elements with an open epiphysis and accelerated bony growth (lipomatous macrodactyly). However, the etiology of tissue overgrowth in macrodactyly is still not clearly understood [1,2].

The term macrodactyly must be reserved for non-syndromic enlargement of digits that occurs singularly without associated limb hemihypertrophy and vascular anomalies, as seen in Proteus syndrome, neurofibromatosis, and other inherited syndromes [1,3]. Here, we present a case of an isolated macrodactyly of the left fourth toe which was treated successfully with debulking surgery. We then discuss a comprehensive review of the literature on the surgical treatment of this difficult and rare condition.

Treatment options can be broadly divided into a form of amputation and digit-preserving surgery by reduction, debulking, osteotomy, and/or physeal arrest (epiphysiodesis). Humphry (1892) first proposed amputation as a treatment option for macrodactyly, especially for cases that severely impede function. Tsuge (1967) proposed surgical preservation of the toe by shortening the digit while simultaneously maintaining both form and function. Although over the years there have been further advancements in debulking or shortening techniques, the results can be disappointing [4].

## Case presentation

A 12-year-old girl presented with enlargement of the fourth left toe since early childhood. The toe was gradually increasing in size over the last two years and the parents were mainly concerned about its appearance. She felt embarrassed about her left foot as it was an object of ridicule among her peers.

**Figure 1:**
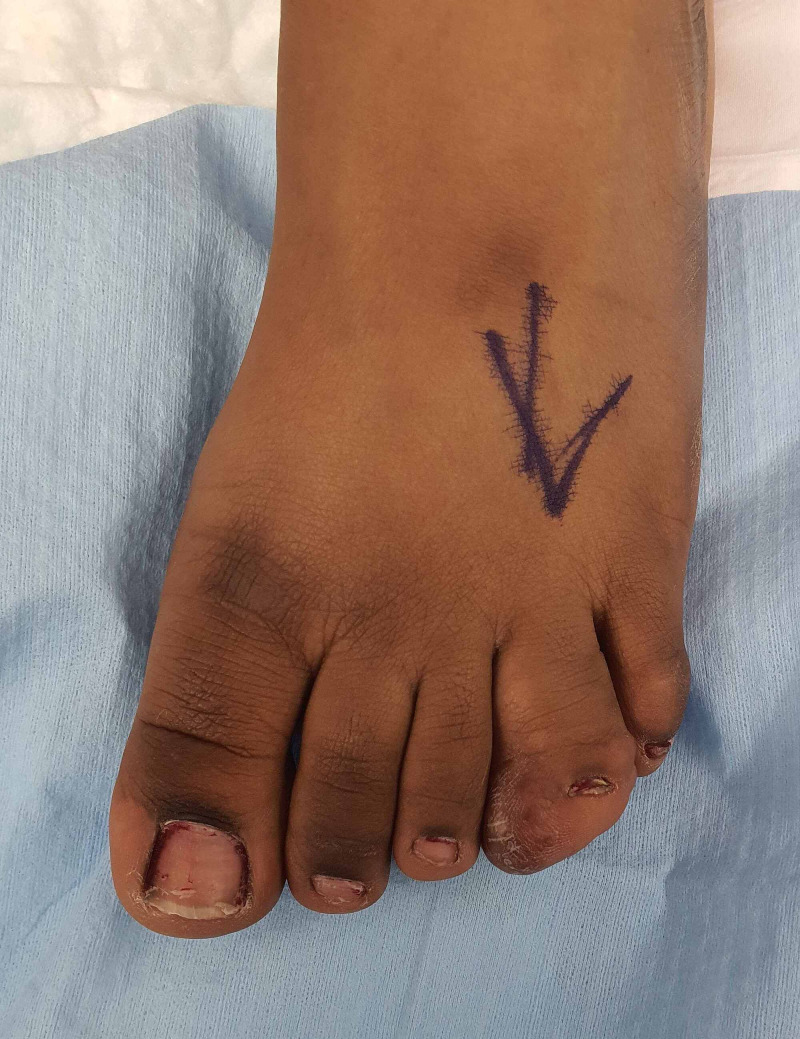
Preoperative image of the toe macrodactyly showing isolated macrodactyly of the fourth toe of the left foot.

On examination, there was a diffuse soft enlargement of the left fourth toe with the nail plate almost buried in the hypertrophied mass (Figure 1). The toe was non-tender to touch, non-pulsatile without any dilated veinsx, and there was no Tinel’s sign on the toe or foot.

**Figure 2:**
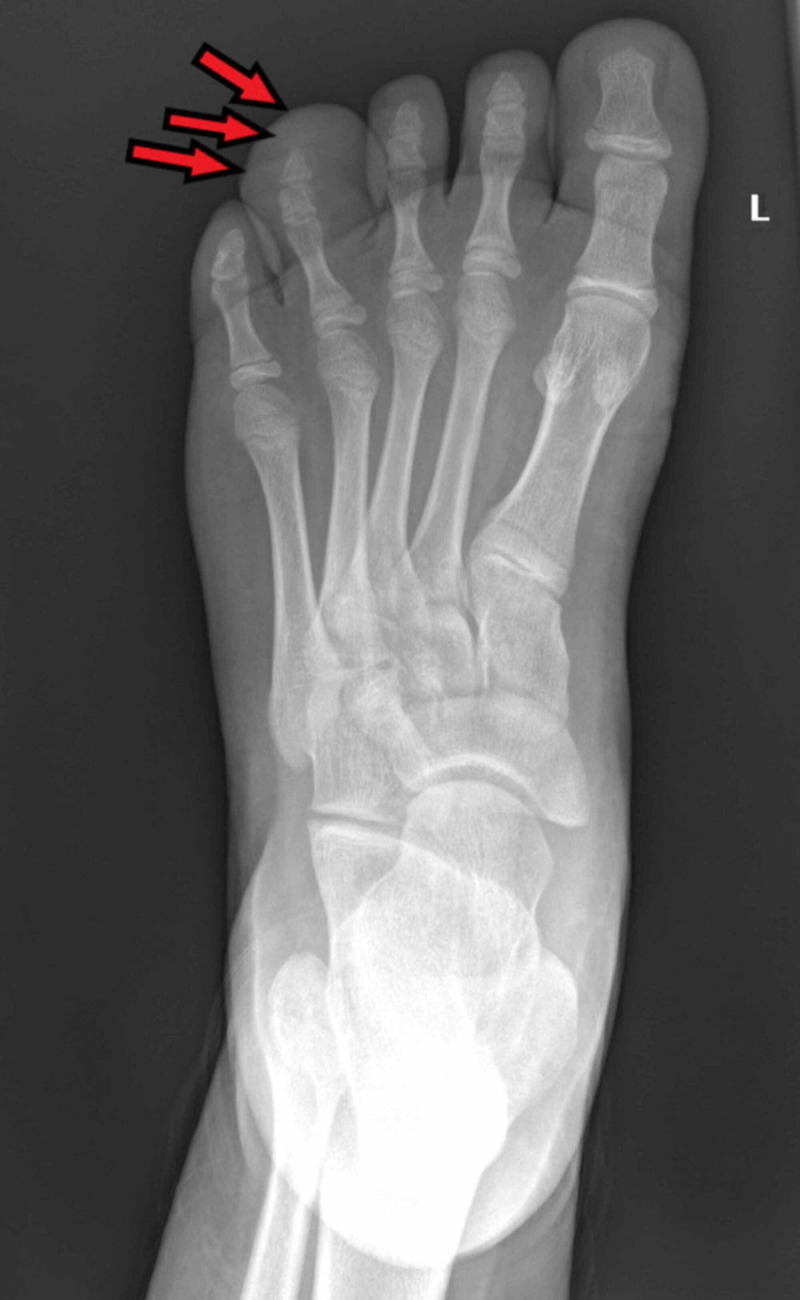
X-ray image of the left foot. Red arrows in the image show the soft enlargement of the affected digit, but the underlying bone is normal with no obvious hypertrophic changes.

X-ray of the affected toe showed no bony abnormality (Figure 2). Ultrasound examination showed diffuse but mildly heterogeneous subcutaneous soft tissue enlargement as well as an increase in vascularity.

**Figure 3:**
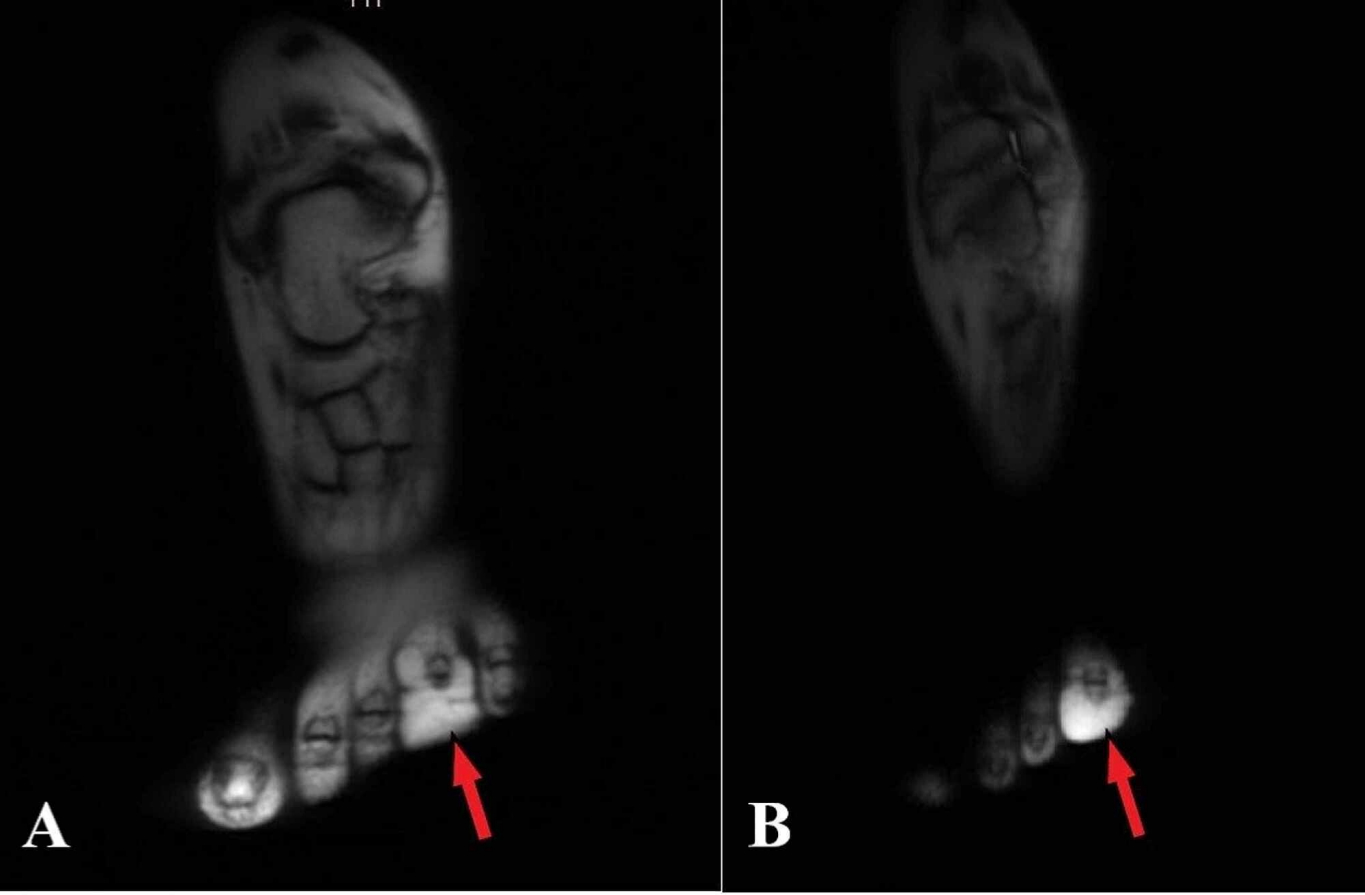
MRI images of toe macrodactyly. MRI shows lobulated large subcutaneous homogeneous soft tissue mass of fat signal intensity with internal fibrous strands located around the left fourth toe (red arrow in A and B).

Subsequent MRI scanning revealed a lobulated large subcutaneous homogeneous soft tissue mass of fat signal intensity with internal fibrous strands around the toe (Figure 3). This was extending from the mid-proximal phalanx to the tip, mainly on the plantar aspect but extending both medially and laterally. No intralesional enhancement was seen.

**Figure 4:**
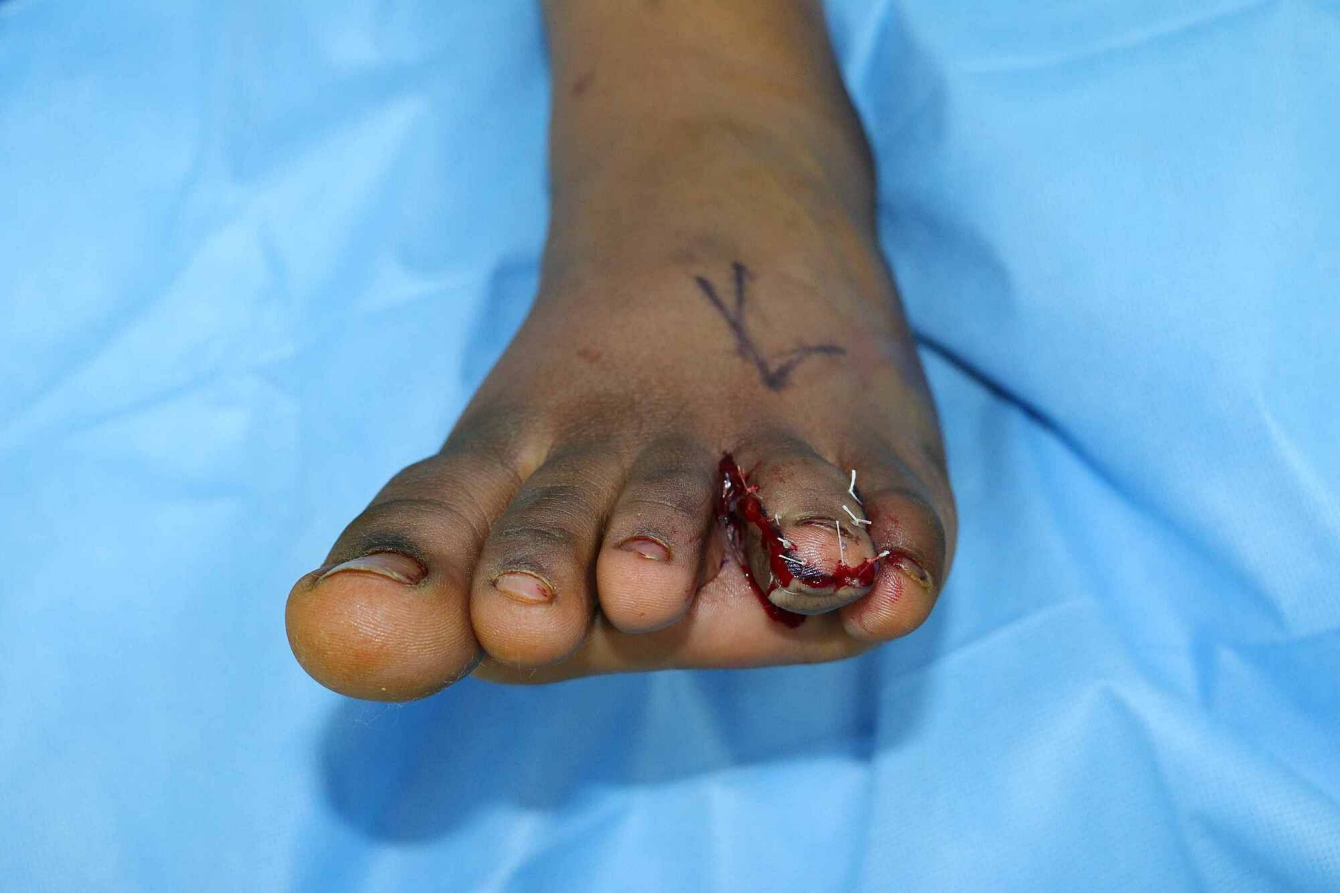
Immediate postoperative image of the left foot showing good reduction of the toe with preservation of both form and function.

The patient underwent the soft tissue debulking procedure under general anesthesia using a long dorsomedial incision to explore the medial neurovascular bundle, which was displaced by abnormal fat in the medial pulp. Removal of the skin and soft tissue was done preserving both the nerve and artery. The incision was extended laterally for further removal of abnormal fat. A physiological amount of normal-looking fat attached to the tip of the distal phalanx was retained to preserve form. Glabrous skin was trimmed adequately and the wound was closed directly without tension (Figure 4).

**Figure 5:**
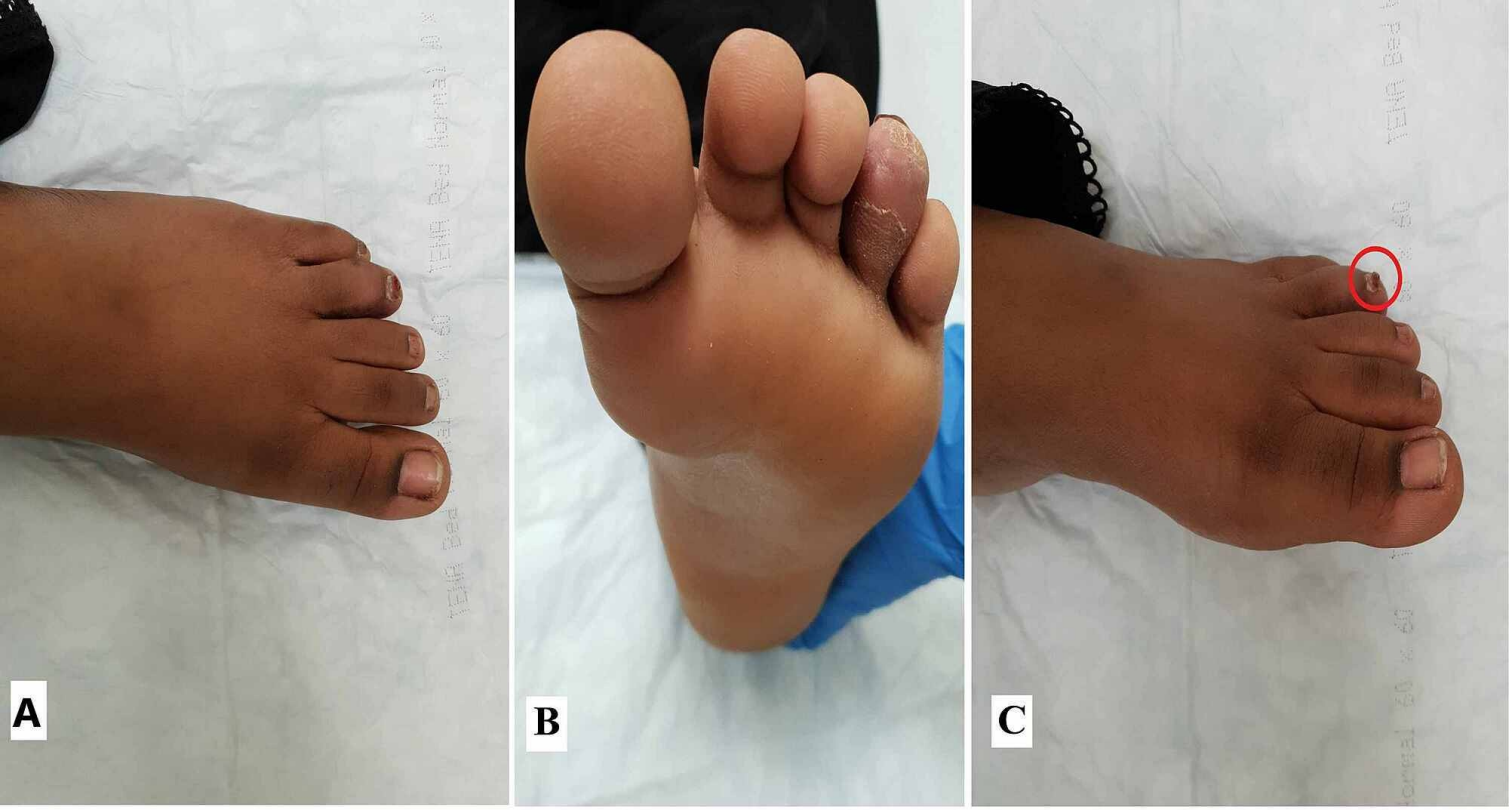
Final postoperative outcomes. Outcome after three months follow-up showing good cosmesis in the dorsal view (A), plantar view (B), and lateral view (C). The red circle in (C) shows an almost normal appearance of the nail plate of the affected toe at three months.

The postoperative course was uncomplicated with discharge from the hospital on the second postoperative day and regular follow-up in the outepatient clinic. She started walking on the day of discharge. There was a lateral deviation of the toe seen immediately postoperatively, which was corrected by a four-week-course of neighbor strapping of the third and fourth toes. The final appearance of the toe at three months was a normal anatomical appearance on the dorsum but with a slightly reduced pulp on the volar side (Figure 5). The sensation on the plantar aspect was preserved with a full range of motion of the toe. Postoperative biopsy report confirmed benign lipohamartomatous overgrowth of the left fourth toe.

## Discussion

The diagnosis of macrodactyly should be solely applied to the presence of isolated enlargement of digit(s). The relative rarity of this condition and lack of a proper, standardized classification scheme has contributed to the paucity of long-term studies in the literature. An astute clinician needs to rule out other syndromic associations of macrodactyly such as Klippel-Trenaunay syndrome, Proteus syndrome, Parkes Weber syndrome, Maffucci syndrome and Ollier disease, hemihypertrophy, lymphangiomatosis, congenital lipofibromatosis, and neurofibromatosis [2,3].

Although the main indication for surgery in macrodactyly is cosmesis, improvement in function is relevant in more severe cases. There is controversy regarding debulking surgery versus amputation in the treatment of macrodactyly. This article aims to shed some light on this issue, but it is clear that in isolated, less severe forms like our case the debulking procedure is far better, whereas in the more extensive macrodactyly cases debulking can often produce a disappointing result.

Preoperatively, there must be extensive counseling of the parents explaining the pearls and pitfalls of surgery and the overall suboptimal results that occur as a result of scarring, stiffness, and the possibility of regrowth necessitating subsequent amputation. Although the ideal time to operate is controversial, it depends on the type of macrodactyly; it is generally agreed that the progressive type should be operated earlier and likely requires further surgery. For other variants, if indicated, surgery should be performed before the child starts walking [4-7].

Another universally accepted principle in the treatment of macrodactyly, the rapidly progressive and severe variant of disease, would be an amputation of the affected digit. The static and milder variants of macrodactyly are generally treated by watchful waiting. However, severe pain, deformity, and regrowth following surgery are relative indications for amputation [4,6,7]. However, in most cases, an initial debulking surgery is always offered to the patient combined sometimes with phalangeal and/or metatarsal shortening with/without epiphysiodesis. Bulut et al. in their case series concluded that a ray amputation is an effective option for severe and recurrent macrodactyly [6].

Few surgical principles must be followed when planning a soft tissue debulking procedure: mid-lateral incision along the digits are preferred, when crossing creases extend as zigzag incisions onto the glabrous skin (to avoid scar contracture), nerve excision can be done as it limits nerve tissue-induced overgrowth, always debulk one side of a digit at a time with proper visualization and preservation of the digital artery, wait for at least three months to debulk the opposite half of the same digit, epiphysiodesis is recommended once the digit approaches the size of the same digit in the parent of the same sex, perform corrective osteotomy for angular deformity at the time of epiphysiodesis, and downsizing of the nail plate and bed is important to achieve a better cosmetic outcome [4-8].

Downey et al. reported two cases of macrodactyly treated successfully with debulking combined with an island nail transfer (onycho-osteo-cutaneous flap) with good cosmesis. Kobraei et al. described reconstruction following nerve resection with nerve allograft and reported improved sensory outcomes [9,10].

Complications reported after surgery include wound dehiscence, infection, prolonged edema, ischemia of wound margins, sensory disturbances, recurrence/regrowth, scar contracture, joint stiffness, and keloid formation. Tolerton et al. have recommended methotrexate therapy to suppress keloid formation and to control recurrence after surgical revision for keloid [4,8,11].

The good result obtained in our case is because it was a less severe, non-progressive variant of macrodactyly where debulking surgery provides consistent and predictable outcomes. However, in the severe, progressive variant, the ideal choice is still a matter of debate. Some surgeons prefer amputation or shortening of the affected digit with/without free nail grafting. Tsuge procedure involves shortening and preservation of the nail unit as a pedicled nail flap done in two stages. Uemera et al. described shortening of the digit and nail preservation using a pedicled vascularized nail graft in a single stage. Hence, it would be prudent to note that the reconstruction of pain-free, mobile, and aesthetically pleasing appearance should be the goal in the treatment of foot macrodactyly [12].

## Conclusions

Macrodactyly is a difficult condition to treat because of its rarity in clinical practice and the overall suboptimal results of surgery. However, despite this, proper counseling for both parents and children, individualized surgical treatment, meticulous surgical planning and execution, and realistic expectations about treatment outcomes would ensure reasonably predictable outcomes with acceptable complication rates.
